# Two plastomes of *Phyllostachys* and reconstruction of phylogenic relationship amongst selected *Phyllostachys* species using genome skimming

**DOI:** 10.1080/23802359.2019.1696244

**Published:** 2019-12-09

**Authors:** Nian-Jun Huang, Jiang-Ping Li, Guang-Yao Yang, Fen Yu

**Affiliations:** Jiangxi Provincial Key Laboratory for Bamboo Germplasm Resources and Utilization, Forestry College, Jiangxi Agricultural University, Nanchang, P. R. China

**Keywords:** Bambusoideae, Arundinarieae, *Phyllostachys reticulata*, *Phyllostachys edulis*, genome skimming

## Abstract

The genus *Phyllostachys* is economically important; however, only a small amount of complete plastid genomes have been reported to date. Here, we characterized two complete chloroplast genomes of *Phyllostachys* using genome skimming. The chloroplast genomes of *Phyllostachys reticulata* and *Phyllostachys edulis* ‘Pachyloen’ were 136,689 bp and 139,678 bp in length, respectively, and their GC contents were 38.8% and 38.9%, respectively. The sequences of each species contained 132 unique genes, including 39 tRNA, eight rRNA, and 85 protein-coding genes. Phylogenetic analysis shows that all selected *Phyllostachys* species were grouped into one well-supported clade in the *Phyllostachys* clade (V) of Arundinarieae. Moreover, in terms of chloroplast genome size, structure, and composition, *P. edulis* ‘Pachyloen’ is identical to *P. edulis*, further indicating the affinity between them.

The genus *Phyllostachys* Siebold & Zuccarini (Bambusoideae: Arundinarieae) comprises at least 51 species and is originally indigenous in China but widely and extensively cultivated in neighboring Asian countries (Li et al. 2006). It is an economically important genus and its species are used for building, paper, flooring, furniture, edible shoots, and as ornamentals.

Information from chloroplast genome sequences has been extensively applied in understanding interspecific relationship (Ma et al. 2014; Li et al. 2019; Zhou et al. 2019). To date, however, only a small amount of complete plastid genomes has been reported for members of *Phyllostachys*, such as *P. propinqua* (Wu and Ge 2012), *P. sulphurea* (Gao and Gao 2016), *P. nigra*, and *P. edulis* (Zhang et al. 2011).

In this study, we reported and characterized the complete chloroplast genomes of *Phyllostachys reticulata* (Ruprecht) K. Koch and ‘Pachyloen’, the latter of which was a new accepted cultivar of *Phyllostachys edulis* (Carrière) J. Houzeau with the certificate number WB-001-2018-028. Fresh leaves were collected from *P. reticulata* in the bamboo garden of Jiangxi Agricultural University, China (28°45′40″N, 115°49′31″E), and from *P. edulis* ‘Pachyloen’ on the type locality Wanzai County of Jiangxi Province (28°20′32.14″N, 114°26′4.07″E). Both voucher specimens were deposited at the herbarium of the College of Forestry, Jiangxi Agricultural University, China (collection numbers are Hnj10253 and Yufen10254, respectively). Illumina paired-end (PE) library was prepared and sequenced in the Kunming Institute of Botany, Chinese Academy of Sciences (CAS) in Kunming, China. Using SPAdes 3.13.0 (Bankevich et al. 2012) and Geneious 9.0.5 (http://www.geneious.com/), all contigs of the chloroplast genome sequence were spliced and assembled. After the annotation of the complete chloroplast genome using the webserver DOGMA (Wyman et al. 2004), simple sequence repeats (SSR) were detected using MISA (http://pgrc.ipk-gatersleben.de/misa).

The complete chloroplast genome sequence of *P. reticulata* (GenBank accession number MN537808) was 139,689 bp in length, and its GC content was 38.8%. LSC and SSC contained 83,221 bp and 12,872 bp, respectively, whereas IR was 21,798 bp in length. The genome contained 132 functional genes, including 85 protein-coding genes, 39 tRNA genes, and eight rRNA genes.

The complete chloroplast genome sequence of *P. edulis* ‘Pachyloen’ (GenBank accession number MN537809) was 139,678 bp in length, and its GC content was 38.9%. LSC and SSC contained 83,212 bp and 12,870 bp, respectively, whereas IR was 21,798 bp in length. The genome contained 132 functional genes, including 85 protein-coding genes, 39 tRNA genes, and eight rRNA genes.

To determine the phylogenetic status of *P. reticulata* and *P. edulis* ‘Pachyloen’, additional 30 complete chloroplast genomes of the trib. Arundinarieae, together with three species as outgroup (Figure 1), were downloaded from NCBI. Using RAxML 8.2.8 (Stamatakis 2014) and MrBayes 3.2.6 (Ronquist and Huelsenbeck 2003), a maximum-likelihood phylogenetic tree and Bayes tree was generated, respectively. Consistent with previous results (Zhang et al. 2019), our results showed that five species and one cultivar of *Phyllostachys* were grouped into one well-supported clade in the *Phyllostachys* clade (V) of Arundinarieae. Moreover, in the terms of chloroplast genome size, structure, and composition, *P. edulis* ‘Pachyloen’ is identical to *P. edulis*, further indicating the affinity between them. In addition, congruent with recent studies (Ma et al. 2014; Zhang and Chen 2016), 11 major lineages of Arundinarieae recovered here is low-supported with short internodes in the ML tree, indicating a probable recent rapid radiation of Arundinarieae.

**Figure 1.**
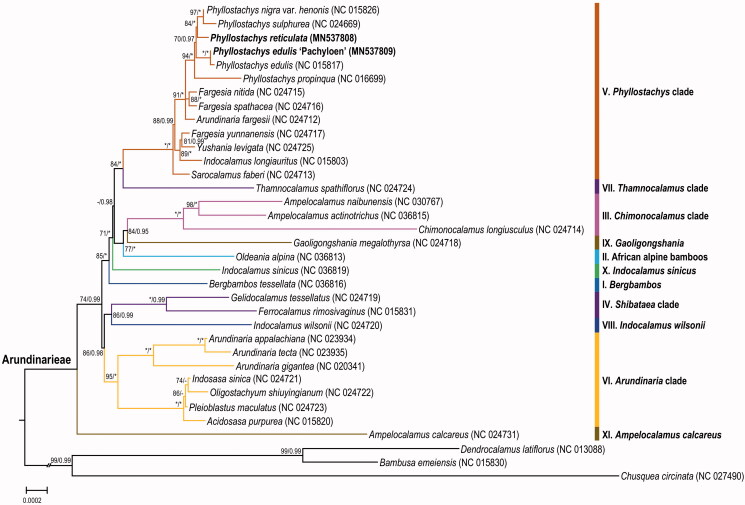
Maximum-likelihood tree inferred from 35 woody bamboo chloroplast genomes. Colored branches indicate the 11 Arundinarieae lineages (I to XI). Numbers associated with branches are ML bootstrap values, and Bayesian posterior probabilities, respectively. Asterisks indicate 100% bootstrap support or 1.0 posterior probability. Hyphens indicate the bootstrap support or posterior probability lower than 50% or 0.5.
